# Observations and perceptions of veterinarians and farmers on heartwater distribution, occurrence and associated factors in South Africa

**DOI:** 10.4102/jsava.v91i0.1763

**Published:** 2020-06-22

**Authors:** Rhoda Leask, Gareth F. Bath

**Affiliations:** 1 Department of Production Animal Studies, Faculty of Veterinary Science, University of Pretoria, Pretoria, South Africa

**Keywords:** climate change, control, diagnosis, epidemiology, heartwater, ruminants, survey

## Abstract

**Background:**

There is currently no scientific evidence regarding the current climatic or other epidemiological factors that could influence the occurrence of heartwater in South Africa.

**Objectives:**

The objective was to determine whether climatic changes or other epidemiological factors influence the occurence of heartwater in South Africa.

**Method:**

A survey was conducted to scrutinise these factors using both veterinarians and farmers working in known areas in which heartwater had previously been confirmed to establish the value of each of these factors. Based on the observations, meaningful tendencies were noted, and conclusions drawn.

**Results:**

These include changes in the spatial distribution of heartwater in many areas, with serious expansion, in some instances, of up to 150 km. In total, 48% of veterinarians and 42% of farmers reported seeing increase in the number of farms affected by heartwater. Climate change as a causative factor indicated by observations of increased average temperatures, milder frosts, less rain and shorter rainy seasons was identified by the majority of farmers but not by as many veterinarians. Respondents in both groups considered vegetation change an important factor. Increasing number of wildlife, especially antelope, was seen as a major factor by most veterinarians and also by many farmers. Both groups identified the movement of livestock and wildlife as an increasingly important factor that should be of major concern for both industries because it leads to the avoidable spread of many diseases apart from heartwater.

**Conclusion:**

Movement controls should be reinstated and reinforced by vigorously enforced legislation. The role of genetically determined resistance or resilience to heartwater infection in ruminants should be investigated. Breeding better adapted animals could provide part of a sustainable approach to the disease.

## Introduction

Tick-borne diseases affect animal and human health and economics worldwide (Estrada-Pena & Venzal 2007; Harrus & Baneth 2005). In South Africa, heartwater, associated with high mortality rates, is caused by an obligately intracellular proteobacterium (Howell, Walker & Nevill 1978) *Ehrlichia ruminantium*, and transmitted by *Amblyomma hebraeum* (Bezuidenhout et al. 1994). It is widely considered as one of the most economically important tick-borne diseases of livestock wherever the natural vector is present. This includes much of sub-Saharan Africa and several Caribbean islands (Allsopp, Bezuidenhout & Prozesky 2004; Curto De Casas & Carcavallo 1995). In South Africa, it is endemic to the north-eastern parts of the country, from the north-east of North West province, through Limpopo and north-eastern parts of Mpumalanga, along the coastal belt of KwaZulu-Natal and the Eastern Cape (Allsopp et al. 2004).

Little data exist on the true economic impact of heartwater, but in 2005 alone losses because of heartwater in southern Africa were estimated around R189.6 million (Spickett, Heyne & Williams 2011). Heartwater is found in extended areas of the north and eastern parts of South Africa, and it is estimated that 35%, 54% and 12% of total cattle, goat and sheep populations, respectively, are at risk of infection (Spickett et al. 2009). The methods presently used to control heartwater are only partially effective. There is no safe vaccine available (Allsopp 2015; Allsopp et al. 2004). Use of prophylactic antibiotics (Allsopp 2009) is costly and may contribute to resistance in the organism. The same could be said about the use of acaricides to control the vector (Allsopp 2009). Breeding for resistance is a long-term goal. Heartwater therefore remains a major obstacle in the potential for productive farming in large parts of South Africa.

The main heartwater vector in South Africa, *A. hebraeum*, is highly adapted to particular climatic and habitat conditions (Allsopp et al. 2004). It is well known that vector-borne diseases such as heartwater are especially sensitive to climatic changes (Harrus & Baneth 2005; Randolph 2010; Slenning 2010). Patterns of continuing global climatic changes associated with anthropogenic greenhouse gases, including carbon dioxide, have been well documented, and there is an expanding bank of evidence of altering weather patterns and geographical changes in vector-borne disease (Slenning 2010). In areas such as Africa where possibilities for livelihood and food production are classified as marginal by the Food and Agriculture Organisation of the United Nations (FAO), it is predicted that even small changes concerning temperature or precipitation and its consequences could be disastrous (Albihn, Gustafsson & O’Hara Ruiz 2012; CurtoDe Casas & Carcavallo 1995). Furthermore, it is expected that developing nations such as South Africa will have less economic and technological flexibility to mitigate effects of climate change than developed nations (Albihn et al. 2012; Allsopp et al. 2004).

In South Africa, habitat suitability modelling based on tick collections of *A. hebraeum* shows the tick to be contained within its historical range (Spickett et al. 2009). However, recent tick surveys in the North West province showed that *A. hebraeum*, which is historically recorded to be present in grassed bushveld and wooded savannah regions of the province, appears to be now well established in mixed and sourish-mixed bushveld areas (Spickett et al. 2011). Another study based on climatic modelling of tick habitat suitability indicates a possible future decrease in habitat structure for *A. hebraeum* depending on temperature fluctuations (Estrada-Pena 2001, 2003).

The epidemiological dynamics of heartwater in South Africa according to available literature is unclear. There is anecdotal evidence from veterinarians, indicating that heartwater distribution is changing, but this has not been formally investigated before. This report tries to provide an indication of whether or not there has been a change in the distribution and incidence of heartwater according to the observations and experience of veterinarians as well as farmers. As subsidiary objectives, it seeks to identify factors, including climate change, that could be associated with a change in the occurrence of heartwater, and to evaluate the potential future impact on livestock production in South Africa.

This investigation was a cost-effective way of using veterinary and farmer knowledge and experience to reveal information on changes, or perceived changes, on heartwater occurrence in a short time. It could serve as a pilot study for further quantitative research targeted to specific areas identified as at maximum risk for the spread or intensification of heartwater.

The objective was to establish whether veterinarians and farmers in heartwater areas of South Africa have seen changes in the distribution and incidence of heartwater, and to identify possible causative factors, including climatic changes, that in their opinion could be associated with any observed changes. Control measures and diagnostic procedures in use were also investigated.

## Materials and methods

The model system was a survey in the form of a structured questionnaire to obtain empirical data from both veterinarians in the field and farmers. Design of the questionnaire was based on the principles published previously, accepted and implemented for use and appropriateness (Bailey 1978; Berdie 1973; Berdie, Anderson & Niebuhr 1986; Geer 1988; McCledon & O’Brien 1988; Montgomery & Crittenden 1977; Sheatsley 1983). The questionnaire was conducted predominantly in electronic form, although a hard copy was sent to those preferring it. The electronic questionnaire was easy to work with compared with postal questionnaires that historically had a low response rate, often below 20% (Holmes & Cockcroft 2008). English and Afrikaans versions of the questionnaire were made available. Missing data were dealt with as described by Dohoo, Martin and Stryhn (2003).

Non-probability sampling was used to make purposeful selection of veterinary practices to be included in the survey (Spickett et al. 2009). Veterinary practices that fell within a habitat-suitability index of 0.3–0.5 for *A. hebraeum* as indicated by recent tick surveys were identified for possible participation (Spickett et al. 2009). The monthly report of livestock diseases on the landbou.com website was used to ensure that all areas that encounter heartwater were included. The ‘Rural Vet’ online discussion group was used as the Internet source to request assistance from livestock veterinarians. Contact with farmers was through farmers’ associations in the areas of the participating veterinarians and media appeals. Preferred veterinary practices were those that diagnosed heartwater in specific areas of the practice only, and where at least one veterinarian had been in full-time service at the practice for a minimum of 5 years.

Of over 120 rural veterinary practices or organisations in South Africa, 74 that were apparently suitable were approached directly to participate in the heartwater survey and 25 volunteered to take part (33%). With the farmers, there were appeals through various media and organisations, so the representativeness of the sample (39) received could not be assessed. Nevertheless, the size of both groups was consistent with the experimental design. The respondents were analysed according to age, gender, length of time in the practice area, area of practice or farm, and percentage of practice activity relating to ruminants. The questions in the questionnaire were both analysable and quantifiable. The questionnaire encompassed various question formats, providing the participant with multiple choice options for single option selection or required the participant to rank or rate options (Holmes & Cockcroft 2008) with the main aim of establishing whether or not there had been a change in heartwater distribution and prevalence as well as the subsidiary aims of establishing the level of possible contributory factors to this change. Possible factors identified included the introduction or increase in wildlife, changes in livestock numbers, type of farming systems used, changes in vegetation, introduction of domestic livestock and changes in weather patterns.

The questionnaire for farmers contained 21 questions and an additional six questions were added to the questionnaire for input from veterinarians. Answers to the questions were returned on ‘SurveyMonkey’ (www.surveymonkey.com) to ensure that all the data were collected at one place. The questions were initially tested on five practices and five farmers to establish clarity and to make any improvements if required. After collection, the data were evaluated for any outliers or inconsistencies in the answers (Holmes & Cockcroft 2008). Each variable was analysed as appropriate in terms of distribution, grouping, mean or range and reported as a proportion of responses to a category (Holmes & Cockcroft 2008). Some data were evaluated further (Holmes & Cockcroft 2008).

### Ethical consideration

Ethical clearance was obtained from the University of Pretoria’s Research and Ethics Committee (reference no. V070-14)

## Results and discussion

The responses of 25 veterinarians were analysed. These were quite well distributed along the known limit of heartwater occurrence, with the notable exception of the far northeastern communal areas of the Eastern Cape province. There were 39 responses from farmers that were suitable for analysis, although the geographic location of many farms was difficult or impossible to establish.

Questions 1–6 were background and qualifying questions.

Question 1: Are there parts of your area where heartwater does not occur?

For veterinarians the area referred to was their service areas, and for farmers it referred to the area they indulge in farming. Veterinarians: *n* = 25; 15 answered yes, 9 answered no and 1 was uncertain. Farmers: *n* = 39; 12 answered yes, 23 answered no and 4 were uncertain. Question 1 established whether heartwater was present in an area and to what extent. Answers that are more satisfactory came from veterinarians, with most (60%) indicating that they operated in or at the edge of the heartwater area, whilst only 31% of farmers indicated that they farmed in or near heartwater areas.

Question 2: Indicate your practice’s magisterial districts/location of your farm.

Veterinarians: *n* = 25, Eastern Cape = 7, KwaZulu-Natal = 6, North-West/Gauteng = 6, Mpumalanga = 3, Limpopo = 3. Farmers: *n* = 36. This established that there was a fair representative distribution of veterinarians throughout the target area, but the situation for the farmers was uncertain.

*Question 3: How long have you been practising*/*living in this area?*

Veterinarians: *n* = 25; periods ranged from less than 5 years (7), more than 5 but less than 10 years (6), more than 10 but less than 20 years (4), to over 20 years (8). Farmers: *n* = 37; periods varied widely from less than 5 years to over 60 years. The results showed a wide, fairly even distribution of time spent in the area for both veterinarians and farmers. In addition, the information on veterinarians, when compared with information obtained in questions 12 and 13, showed that the time in practice had negligible influence on the responses given in the latter questions.

Question 4: How many years of experience do you have with treating/working with ruminants?

Veterinarians: *n* = 25, ranging from 3 to 40 years with a mean of 17 years. Farmers: *n* = 37, also ranged widely from 5 to 50 years. This information supported the data in question 3 and indicated that the great majority of both groups had sufficient experience with ruminants to make their input to the survey valuable.

Question 5: How many veterinarians are currently employed at your practice?

Numbers given varied from 1 to 9 veterinarians per practice: 1-person practices = 8 (36%), 2-person practices = 3 (14%), 3–5 persons = 5 (23%) and more than 5 persons = 6 (27%). Three of the 25 responded with ‘State Vet’, which could indicate single or multiple veterinarians. These results revealed a mean of 2.7 veterinarians per practice, with 29% of practices employing more than three veterinarians.

Question 6: Approximately what percentage of the practice caseload are ruminants?

Two veterinarians of the 25 did not respond to the question: *n* = 23; 0% – 20% = 5 (22%), 21% – 40% = 2 (9%), 41% – 60% = 8 (35%), 61% – 80% = 4 (17%), and 81% – 100% = 4 (17%). The case load of ruminants for practices indicated good exposure, with most (69%) showing a 41% or more share of ruminant work.

Question 7: What is the approximate number of farms per year the practice provides service to?

Two of the 25 veterinarians did not respond to the question: *n* = 23, 0–20 farms = 4 (17%), 21–40 = 9 (39%), 41–60 = 4 (17%), 61–80 = 1 (4%), and 80 or more farms = 5 (22%). The results showed good involvement with farms, with 43% reporting activity on 40 farms or more.

*Question 8: Rate the importance of heartwater in your area on a scale of 1–10* (Table 1).

**TABLE 1:** Rating of importance of heartwater in the veterinarians’ or farmers’ areas.

Category	Minor importance (1–3)		Intermediate importance (4–6)		Major importance (> 7)	
	*n*	%	*n*	%	*n*	%
Veterinarians *n* = 25	4	16	3	12	18	72
Farmers *n* = 30	2	7	3	10	25	82

Note: Some farmers did not complete the question.

Table 1 shows that the vast majority of veterinarians and farmers rated heartwater as of major importance.

Question 9: Have you noticed any changes in the number of heartwater cases in your area?

- Changes in spatial distribution were only answered by veterinarians: *n* = 25. Fifteen veterinarians (60%) saw no changes in the distribution of heartwater, nine (36%) experienced a less than 50% increase and one veterinarian (4%) reported an increase of over 50%. No respondents experienced any decrease in the distribution of heartwater in their areas.
- Changes in the number of heartwater cases seen. Although the majority of veterinarians (60%) and a significant number of farmers (45%) experienced no change, there was a substantial number of both (40% and 35%, respectively) that were examining an increase in the numbers of cases, with very few or no respondents experiencing decrease. This indicates that current control is failing.
- Change in the number of farms affected (Table 2). The numbers in Table 2 indicate a definite spread of heartwater in many areas.

**TABLE 2:** Changes observed in the number of cases seen and farms affected.

Category	Decrease in number of cases (> 50%)		Decrease in number of cases (< 50%)		Constant/No change		Increase in number of cases (< 50%)		Increase in number of cases (> 50%)	
	*n*	%	*n*	%	*n*	%	*n*	%	*n*	%
**Cases seen**										
Veterinarians *n* = 25	0	-	0	-	15	60	9	36	1	4
Farmers *n* = 31	3	10	3	10	14	45	10	32	1	3
**Farms affected**										
Veterinarians *n* = 25	0	-	0	-	13	52	11	44	1	4
Farmers *n* = 31	2	11	1	5	8	42	8	42	0	-

Question 10: Have you noticed any changes in the occurrence of heartwater recently?

Veterinarians (*n* = 25): None saw decrease only, two veterinarians (8%) saw both increase and decrease, 10 (40%) saw only increase, whilst 11 (44%) saw no change and two veterinarians (8%) were not sure. Farmers (*n* = 31): Two farmers (6%) saw only decrease, four (13%) saw both increase and decrease, eight (26%) saw only increase, 12 (39%) saw no change and five (16%) were not sure. This shows a similar pattern for recent changes in occurrence: Most veterinarians (44%) and farmers (39%) report no change in occurrence, whilst 40% of veterinarians and 26% of farmers reported that they saw only increase in occurrence. In the short-term impressions elicited by this question, this is another cause for real concern about the effective control of heartwater.

Question 11: Indicate which months of the year are the worst months for heartwater?

**FIGURE 1:**
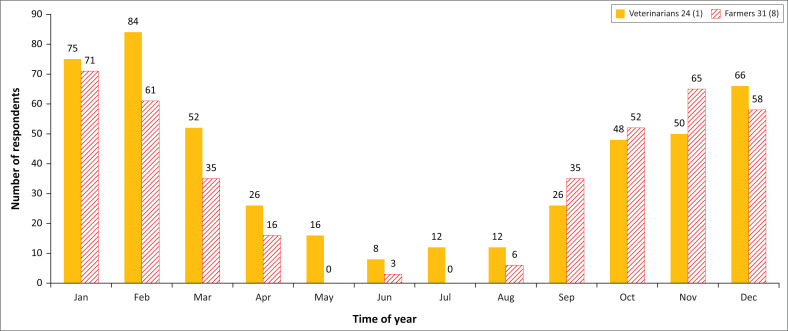
Months of the year where heartwater incidence appears to be the highest.

Veterinarians (*n* = 24) and farmers (*n* = 31). Figure 1 indicates the monthly distribution pattern seen by both veterinarians and farmers which was very similar and in agreement with other reports.

Question 12: In which ‘out of season’ months does heartwater occur?

As expected, these results are largely a reciprocal of the bar graph to question 11 (Figure 1). However, only 16 veterinarians (64%) responded, and four (16%) reported an all-year-round occurrence. With the farmers, only 10 answered the question (26%). Like veterinarians, most reported April to September as the months when heartwater had been encountered unexpectedly. In response to a secondary question directed only at veterinarians to identify reasons for changes in annual incidence, 10 out of 25 (40%) did not respond; eight (32%) identified climate change, especially warmer winters (4, or 16%), whilst three (12%) said it was non-seasonal. Ticks, fire, game and management were also identified once each as causes of change in annual incidence.

*Question 13: How does the current monthly occurrence of heartwater compare with previous years?* (Table 3).

**TABLE 3:** Opinions on change in heartwater occurrence compared with previous years.

Category	Fewer months at risk		More months at risk		No change		Uncertain	
	*n*	%	*n*	%	*n*	%	*n*	%
Veterinarians *n* = 25	1	4	5	20	13	52	6	24
Farmers *n* = 31	3	10	7	23	10	32	11	35

The majority of farmers and veterinarians reported no change or uncertainty as seen in Table 3.

*Question 14: On a scale of 0–5, rate the importance you believe each of the following factors has on any change in the seasonal distribution of heartwater* (Table 4).

**TABLE 4:** Rating of factors that could contribute to changes in the occurrence of heartwater.

Factors	Respondents				Importance score from 0 (unimportant) to 5 (extremely important)					
	Category	Responses		Skip	0–2		3–4		5	
		*n*	%		*n*	%	*n*	%	*n*	%
Changes in weather	Vets	22	88	3	9	41	13	59	6	27
	Farmers	26	67	13	3	12	23	88	15	58
Changes in vegetation	Vets	22	88	3	7	32	15	68	2	9
	Farmers	26	67	13	7	27	19	73	9	35
Movement Wildlife or livestock changes	Vets	23	92	2	2	9	21	91	6	26
	Farmers	25	64	14	1	4	24	96	11	44
Management changes	Vets	23	92	2	4	17	19	83	5	22
	Farmers	26	67	13	3	12	23	88	15	58

A small number of veterinarians and farmers made some additional comments without a clear pattern emerging. Factors included climate, stock movement, sale of undipped animals, new farmers, lifestyle farming, more sheep or goats, and new strains of the organism.

*Question 15: Indicate all the prevalent antelope wildlife (free-living and ranches) in your area* (Table 5).

**TABLE 5:** Prevalence of antelope wildlife and ranking of importance from 1 being most important to 7 being least important in the spread of heartwater ticks.

Wildlife species	Responses and rankings					
	Veterinarians (*n* = 25)			Farmers (*n* = 27)		
	Responses		Ranking	Responses		Ranking
	*n*	%		*n*	%	
Kudu	22	88	1	18	67	1
Wildebeest	21	84	2	7	26	4
Blesbuck	20	80	3	18	67	2
Impala	20	80	3	14	52	3
Giraffe	11	44	5	6	22	6
Springbuck	10	40	6	4	15	7
Sable	9	36	7	7	26	4

A small number of respondents provided a long list of other wildlife without revealing a useful indicator of species that may play a role in the spread of heartwater. There was a good agreement between veterinarians and farmers on the potential role in spreading heartwater ticks by kudu and impala, which are both excellent at jumping fences. Reports of the presence of other antelope clearly demonstrate the possible role of wildlife in maintaining tick populations. It was of interest to note how often giraffe and sable were recorded by both respondent groups.

Question 16: Which of the following intermediate hosts for heartwater ticks are present in your area?

Table 6 shows the presence and rankings of intermediate hosts for heartwater on farms surveyed.

**TABLE 6:** Presence and rankings (1 most important to 7 least important) of intermediate hosts for heartwater in the areas surveyed.

Wildlife species	Responses and rankings					
	Veterinarians (*n* = 25)			Farmers (*n* = 28)		
	Responses		Ranking	Responses		Ranking
	*n*	%		*n*	%	
Guinea fowl	25	100	1	26	93	1
Bush pig	21	84	2	14	50	5
Rodents	20	80	3	23	82	2
Scrub hare	19	76	4	19	68	3
Warthog	16	64	5	15	54	4
Tortoise	16	64	5	14	50	5
Cattle egret	10	40	7	9	32	7

Whilst six veterinarians and 13 farmers listed other species, these were considered negligible in their role in the spread of heartwater. Known intermediate hosts for immature stages of heartwater ticks or other carriers of ticks were indeed abundant so that the tick once transferred to new suitable environments could readily be established permanently.

Question 17: In your opinion have any of the following factors changed in your area?

The results to questions 17 and 18 are discussed in more detail below.

Question 18: Rate the importance on a scale of 0 (of great importance) to 11 (of no importance) the effect on the occurrence of heartwater of each of the following factors.

On the factors of weather and climate, the survey revealed the greatest differences between the two groups. Regarding average temperatures, 54% of veterinarians saw no change, whilst 43% of farmers saw gains. Regarding rainfall, most veterinarians (61%) reported no change, whilst most of farmers (54%) reported a decrease. Regarding the length of rainy season, most veterinarians (38%) saw no change, whilst most farmers (60%) reported a decrease. Finally, regarding frost, most veterinarians (58%) saw no change, whilst with farmers there were equal responses for ‘no change’ (31%) and for increased severity of frost. When this response is related to the information given for weather and climate in question 15, there is consistency in the replies given in both groups. However, significant discrepancies between veterinarians and farmers on the importance of climate change were evident, with an average ranking of 6 for veterinarians and a higher ranking of 4 for farmers in question 19. Regarding vegetation and bush cover, the veterinarian group was more consistent. This aspect was given similar importance in question 15 with the majority (58%) saying that bush cover was increasing. It was rated as number 4 in the list of importance. The farmer group rated it higher in question 15, yet were evenly split between increase (33%) and decrease (33%) and only rated it as a low factor 9, revealing an important degree of inconsistency.

Regarding the presence of wildlife as a factor, veterinarians saw this as very important, with 79% reporting increase and ranked it as number 1. Farmers rated this as far less important, with 46% finding increase in wildlife and ranked it a much lower 6.

The groups also differed in observations about the use of heartwater vaccine: 40% of veterinarians reported a drop in use, whilst 52% of farmers reported no change. Both groups ranked this aspect at low 8 and 9, respectively.

Tick control was seen as a less important factor by veterinarians, with 36% reporting ‘no change’ closely followed by ‘decrease’ (32%), but ranking it as 3. The farmer group mainly reported an increased use of dipping (36%) followed closely by ‘no change’ (32%), yet ranked dipping as a very high factor at 1. Neither group was thus very consistent in their answers.

Regarding prophylactic blocking, both groups believed that there was a major increase in the use of this control method. Of the veterinarians, 44% saw increase with 32% reporting no change, ranking it as factor 5. In the farmer group, 36% saw increase, whilst 32% reported no change, giving it as factor 1.

Finally, management was seen as an important factor in both groups. However, there could have widely diverging interpretations of what was meant by ‘management’.

Questions 19–21 covered aspects of diagnostics of heartwater and are discussed below question 21.

Question 19: Which of the following diagnostic methods have been used in support of the diagnosis of heartwater?

Question 20: What percentage of suspected heartwater cases or outbreaks is confirmed by a brain smear or other laboratory diagnostic methods?

*Question 21: Which of the following laboratory diagnostic methods have you used to confirm the diagnosis of heartwater?* (six options were supplied; veterinarians only).

All 24 veterinarian respondents listed standard brain smears. In addition, one used immunoperoxidase staining because of easy access to the technique. None of the other options listed were used.

The above questions addressed diagnostic approaches used for heartwater. There were clear and important differences between the groups: veterinarians used a wider diversity of methods in a more balanced way than farmers who relied mainly on clinical signs, course of the disease, tick presence and treatment response. Veterinarians relied much more on confirmatory evidence than farmers, who reported that 77% only confirmed heartwater in less than 20% of cases. The scope for misdiagnosis is obvious, especially because tetracycline has a very broad spectrum activity and is used in curing many diseases other than heartwater. It was clear from responses that alternative methods of diagnosing heartwater have had virtually no impact, and ways of reliably confirming provisional diagnosis are lacking (or are not in use for living cases). There were relatively few reports of cases of atypical heartwater. It was perhaps significant that some of these forms were only reported by single respondent veterinarians. It is unlikely that certain forms of heartwater only occur in one region, and more likely that unusual cases are either overlooked or misdiagnosed as something else.

*Question 22: Have you noticed any atypical clinical signs in heartwater cases? Please specify and indicate how many of such signs have you seen?* (veterinarians only).

Only 10 veterinarians responded. Atypical forms described comprised the following: peracute cases – no symptoms (3); abnormal gait (stiff, lame) (2); cold heartwater – no fever (1); neurological cases – not specified (1); severe haemorrhagic enteritis (1); atypical forms unknown (2).

*Question 23: Please share any additional comments*.

Only three veterinarians provided additional comments. One said that heartwater is one of the greatest problems in the Eastern Cape. Another emphasised the severe long-term risk of anti-microbial resistance involved in the widespread practice of blocking the entire herd or flock with tetracycline every 10–14 days for long periods. The third veterinarian questioned the assumption that climate change was to blame, citing his own records going back decades that did not reveal significant changes in weather patterns. Fourteen farmers responded, largely giving their own ‘formulae’ for controlling heartwater, involving blocking, intensive dipping, using an indigenous breed and other observations.

## Conclusion

The survey, although limited in scope, achieved the aims set out for the project. The sample size, structure, demographics, geographic distribution and experience profiles of both veterinary and farmer groups who participated were adequate for gathering useful and reasonably reliable data and the conclusions drawn.

It is concluded from the responses that there has been epidemiological changes in the spatial distribution of heartwater in many areas, with serious geographic expansion in some. There are reports of expansion of up to 150 km, and 48% of veterinarians and 42% of farmers reported discovering increase in the number of farms affected by heartwater, which underlines the conclusion drawn.

Many factors were identified as playing some role in causing these changes. Although impact assessments differed between the respondent groups, there was some agreement that the factors identified had changed, at least in many cases, and that these changes had affected the distribution and/or severity of heartwater. Climate change, indicated by observations of increased average temperatures, milder frosts, less rain and shorter rainy seasons, was seen by the majority of farmers as an important factor that had affected occurrence of heartwater. However, many veterinarians did not share this view. Changes in vegetation, not necessarily caused only by climate change, were considered important factors, presumably in improving habitat suitability for the tick vector, by some respondents in both groups.

An increasing presence of wildlife, especially some antelope species, was seen as a major factor by most veterinarians and many farmers in the increased effect of heartwater. Because these species can harbour the heartwater organism or carry ticks, they could also constitute a reservoir for it. Both groups identified the movement of both livestock and wildlife as an increasing and important factor in spreading the disease. Movement control does not appear to be sufficient or satisfactory.

The future impact of relying on tick control must be of great concern because more frequent dipping of whole herds or flocks to achieve control of heartwater must eventually lead to the development of severe acaricide resistance. Control achieved by routine and regular block treatments of entire flocks or herds was also seen as a major factor and as increasing in use for both respondent groups, each giving it a high ranking. However, reliance on blocking must lead to a much greater risk of developing widespread antimicrobial resistance against the one drug group (tetracyclines) that is cheap and currently very effective in treating heartwater. There is anecdotal support that resistance to this drug has already begun to emerge. It is clear that satisfactory heartwater control could only be achieved by concerted, balanced and epidemiologically sound management and not by unsustainable and ultimately dangerous reliance on total acaricidal tick control and routine suppression of infection by antibiotics.

Heartwater diagnosis at necropsy, backed by appropriate histopathological staining and examination, is both reliable and accurate. However, few farmers confirm suspected cases by means of laboratory testing which may result in misdiagnosis and a true reflection of prevalence of the disease is thus difficult to obtain. Another complication in the diagnosis of the disease is the lack of typical clinical signs, which is confounded by atypical forms of heartwater.

## Recommendations

As this survey was based on perceptions, experiences and opinions of two limited, though representative groups of volunteer respondents, the results should be checked and verified by further more extensive, comprehensive and objective investigations. In particular, changes in geographic distribution and severity of heartwater require additional verification. The role of factors identified in this survey, including possible changes in climate, vegetation cover, wildlife presence and movements of livestock require further investigation.

Development of a true vaccine that is practical, effective, safe and affordable should be of the highest concern and priority. After decades of trials, researchers at Onderstepoort Veterinary Institute have developed a very promising candidate vaccine; its further development to commercial stage by Onderstepoort Biological Products must receive more urgency and attention.

Because the diagnosis of heartwater in living animals rests too much on clinical signs that are not ranked by importance, reliability, regularity and severity, the chances of misdiagnosis should be clear. Research should be conducted to improve the reliability of a diagnosis of heartwater in live animals in terms of a weighted checklist or useful laboratory tests or both.

The role of genetically determined resistance or resilience to heartwater infection in ruminants should be investigated. Breeding better adapted animals could provide part of a sustainable approach to the disease.
